# Not All Canadian Cancer Patients Are Equal—Disparities in Public Cancer Drug Funding across Canada

**DOI:** 10.3390/curroncol29030166

**Published:** 2022-03-17

**Authors:** Ceilidh MacPhail, Stephanie Snow

**Affiliations:** Medical Oncology, Department of Medicine, Dalhousie University, Halifax, NS B3H 2Y9, Canada; stephanie.snow@nshealth.ca

**Keywords:** oncology, universal health care, drug funding, Canada

## Abstract

Canada lacks a national drug insurance plan. The home province or territory of a patient determines which cancer drugs are available on the public formulary, who is eligible for public coverage and what portion of the financial burden of cancer care falls to the individual. This narrative review describes the current interprovincial disparities in access to cancer drugs across Canada. Health technology assessment (HTA) of drugs at a provincial and territory level is a closed process, does not necessarily follow the recommendations of national HTA and leads to further delays in drug access. The public coverage of take-home cancer drugs (THCDs) in Ontario and the Atlantic provinces is often fragmented, unnecessarily complex and a barrier to cancer drug access. Policy solutions to address inter-provincial formulary variation and poor access to THCDs are discussed.

## 1. Introduction

The Canadian health system was founded with the Canada Health Act (CHA) enshrining five criteria for health care delivery across the nation. Healthcare is to be publicly administrated, accessible, comprehensive, universal and portable [1]. The provinces and territories have been entrusted with delivering healthcare, meeting each of these criteria. Provinces and territories determine which cancer drugs are added to the public formulary, which introduces variation in access.

While Canada has a universal health system, there is no accompanying national drug insurance plan. Due to the CHA, cancer drugs given within the hospital environment, such as intravenous (IV) chemotherapy, are publicly covered. IV treatments are also publicly covered when delivered by cancer agencies. In contrast, large disparities exist when it comes to the public coverage of take-home cancer drugs (THCDs), the majority of which are oral medications. Whether THCDs are accessible to a cancer patient is determined by an individual’s home province, income, access to private drug insurance and demographics [1,2,3]. A large burden of the costs for THCDs frequently falls to the patient, leaving them vulnerable to medication non-adherence, or even rendering effective medications inaccessible [4].

There are already significant disparities in the risk of developing cancer, accessing diagnostic and cancer care services and resultant cancer outcomes in Canada on the basis of socioeconomic, geographic and ethnic variabilities [5]. Interprovincial variations in the access to cancer drugs exacerbate existing health inequalities and further contribute to disparities in cancer outcomes across Canada. This narrative review aims to describe the disparities in cancer drug access across Canada. We focus on the health technology assessment process and the differences across provincial and territory public drug plans. While this is not a new policy discussion, the growing use of targeted THCDs in oncology calls for efforts to address inequities in drug access. With THCDs accounting for 45% of national cancer drug sales [6], solutions are needed now.

## 2. Methods

This narrative review of the literature was conducted by searching the terms “Oncology”, “Drug Access”, and “Inequality” on PubMed in October 2021. Articles published in English and after the year 2001 were included in the review. A 20 year window was selected by authors to screen for articles relevant to the current Canadian health system. Titles of articles in search results were reviewed and articles that appeared relevant were then selected for abstract review and subsequently full text review. Snowball referencing was employed for articles included in this review. To capture the grey literature, the above search terms were entered into the Google search engine. Territorial and provincial drug formularies and insurance plans were reviewed to provide details regarding public drug coverage.

## 3. Drug Assessment and Approval to Public Formulary

**Figure 1:**
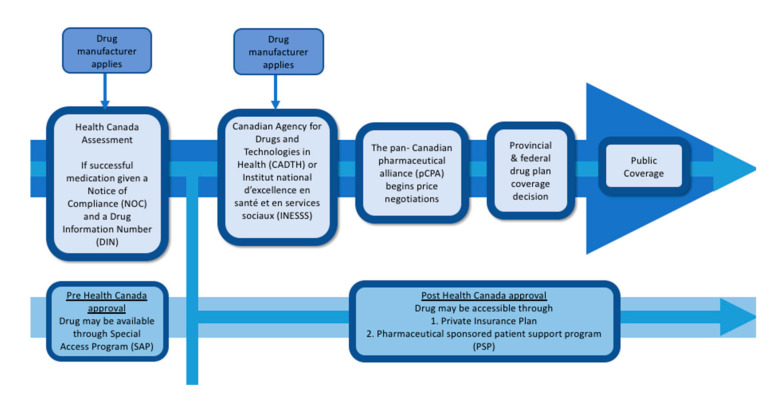
Institutions involved in drug assessment and addition to public formulary in Canada.

Canada has a complex process for drugs to enter the national market and potentially be accessible through public funding. Each new medication must be assessed for efficacy, undergo a health technology assessment, a pricing negotiation and lastly provincial, territorial or federal insurance plan funding approval (Figure 1).

Health Canada first reviews a drug’s efficacy and safety data before granting a notice of compliance and a drug information number (DIN) [7]. With a notice of compliance, a new drug may be accessible through private insurance plans and patient support programs.

The pan-Canadian Oncology Drug Review (pCODR) Expert Review Committee, which is an appointed pan-Canadian advisory body to the Canadian Agency of Drugs and Technologies in Health (CADTH), and the Institut National d’Excellence en Santé et en Services Sociaux (INESSS) then perform a drug assessment, with the latter serving only Quebec. These two organizations independently review a medication from medical and economic perspectives and then make a recommendation for public funding. INESSS and CADTH may arrive at different decisions regarding public funding for a single indication. Generally, after CADTH and INESSS make a recommendation for coverage, the Pan-Canadian Pharmaceutical Alliance (pCPA) will begin negotiations to set the price of a medication with the manufacturing company. This is a closed process and the negotiated price is not made public [8].

Each province and territory independently decide if a drug will be added to their public formulary. It should be noted that certain populations have federal insurance coverage, including those of First-Nation and Inuit descent who are eligible for the Non-Insured Health Benefits (NIHB) plan and public servants who are covered on the public service health care plan [9,10]. For each public plan, the decision to fund a cancer drug is influenced by CADTH or INESSS recommendations, local interests, and financial constraints [9,11,12,13].

How jurisdictions make the decision to add a drug to public formulary is not well studied. A 2005 questionnaire sent to each provincial Drugs and Therapeutics Committee explored the differences in committee structure and decision-making process. [1] Physicians constituted 31–60% of seats, pharmacists 17–25% of seats and the remaining seats were held by administrators. Two jurisdictions, not identified by the authors, reported lacking a standard process. When asked about factors influencing committee decision making, all jurisdictions rated evidence of drug efficacy and budgetary impact highly. Promotional literature and testimonials were considered low in importance. Nevertheless, there was heterogeneity in the types of information considered by the provinces.

Srikanthan and colleagues investigated reasons for discordance in public funding of cancer drugs and pCDOR recommendations by interviewing the policymakers involved in these decisions [12]. They reviewed pCODR recommendations regarding 64 cancer drugs. Reasons for why drugs recommended by pCODR were not funded included budget constraints (90.9% respondents) or disagreement with clinical or economic review. Prince Edward Island (PEI) has been highlighted as having a higher degree of discordance with drug coverage and pCODR recommendations [5].

Beyond the differences of which drugs each province decides to fund, there is variation across Canada in how long each jurisdiction takes to add a new cancer drug to the public formulary. In 2017, a study found the introduction of the pCODR process reduced the time from drug assessment by Health Canada to first public funding from 522 to 363 days [14]. Gotfrit et al. studied the timeline for 21 drugs treating advanced colon, lung and breast cancer to reach the public formulary between 2011 and 2016 [15]. The median time from Health Canada assessment to first public funding was 26.6 months. There was considerable variation, however, among the different provinces in time to approval. For instance, there was a 17.5-month difference between the first and last province to approve pertuzumab for advanced HER2 positive breast cancer, and 20.7-month difference for approval of maintenance pemetrexed used in metastatic lung cancer. The same applies to THCDs; osimertinib, which received a recommendation for public funding by pCODR in 2017, was added to the public formulary by British Colombia (BC) in 2018, to NLs formulary in 2020 and remains unfunded in PEI [5].

Gotfrit et al. used the time taken for drugs to reach the first provincial formulary funding to estimate the life years lost during this process. While there are a number of assumptions made in their estimate (number of patients with lung, breast and colon cancer, and reported overall survival from phase III trials), they found 39,067 life years lost over 2011–2016. It would be a logical presumption that this burden of life years lost would disproportionately impact those provinces where time to funding is the longest.

Another barrier to cancer therapy access is the duration of drug approval. In Quebec, an exception status cancer drug is approved for 4 months, Ontario for 6 months and Nova Scotia for 12 months. The renewal of coverage usually requires the completion and submission of paperwork and radiography to document the response to treatment. Despite 61% of oncologists reporting access to a drug access navigator, a survey of medical oncologists across Canada found 18% were spending 10 h or more per week seeking drug coverage for patients [16].

## 4. Variation in Public Coverage of Take-Home Cancer Drugs

Alberta, BC, Saskatchewan and Manitoba have created cancer agencies whose mandates include administration of cancer drugs. A patient diagnosed with cancer within these provinces, assuming they have a valid health card, meets indications for treatment and are followed by an oncologist, is then able to access a publicly covered THCD. Manitoba’s cancer agency requires patients to be registered with pharmacare and then apply to the cancer agency, but then fully covers cancer drugs on formulary. Quebec requires all residents to have drug coverage be it private or public. As such in Quebec, TCHD coverage may vary by an individual’s drug plan (Table 1).

In contrast, patients in Ontario and the Atlantic provinces must navigate a patchwork of drug coverage [17]. Public drug insurance plans in Atlantic provinces are structured to support patients on social assistance or age 65 and older. Ontario’s public coverage includes individuals over age 65 and under age 25 and those on social services. A recent study conducted by the PDCI Market access [18] and highlighted by the advocacy group CanCertainty [19] found that 17–30% of cancer patients aged 25–64 in Ontario have no form of drug coverage. Paying out-of-pocket is not a financially sustainable option for most Canadians, with average cost of THCDs estimated at CAD 6000 per month [19]. Patients without public or private drug coverage, or after exhausting their private coverage, will need to apply to provincial insurance plans for catastrophic drug coverage.

Applying to a provincial insurance plans requires time, and effort to prepare financial statements documenting household income and the costs of cancer treatment [20]. Tax documents used to assess eligibility for public coverage may not accurately reflect income, due to lost earnings of the patient or caregivers since the cancer diagnosis. A survey of Canadians living with cancer reported confusion when applying to public plans and anxiety regarding the delays in coverage [21].

Ontario and Atlantic provinces have developed catastrophic drug plans to support patients facing high drug cost due to cancer or other conditions. In Nova Scotia, a patient under age 65 requiring support for THCDs can apply to Family Pharmacare. A patient must disclose their family income to determine their copayment and deductible rates. The province will only fully cover a THCD after a patient has spent the pre-determined copayment and deductible. For a household making CAD 65,000 per year with two dependents, out-of-pocket costs totaling CAD 5900 would be required before full coverage begins [22]. The majority of THCDs in Nova Scotia require exception status application by a prescribing physician, which can further delay access. In 2018, Nova Scotia created the TCHD fund, for patients spending 4% or more of net family income. This fund was a short-term solution with funding budgeted for only three years [23]. Ontario offers catastrophic drug coverage to patients aged 25–64 through the Trillium program. This program also requires 4% or more of after-tax income to be spent on drugs before an individual is eligible to apply.

A recent report reveals the discrepancies in public coverage of THCDs across Canada [6]. The report detailed 2019 THCD sales revealing 59% of sales in Nova Scotia, New Brunswick, NL and PEI were covered with a private insurance plan or out-of-pocket [6]. In Ontario, 36% of THCDs were covered privately, whereas private THCD coverage in Alberta was only 12%. Even when public or private insurance is in place, the patients’ share of the cost through co-pays and dispensing fees are frequently as high as 20%. Atlantic Canadians are at increased risk of financial toxicity with THCDs.

Public coverage for supportive care medication also varies by jurisdiction. One example is pegfilgrastim, a drug used to prevent febrile neutropenia. Pegfilgrastim is covered in Ontario and Saskatchewan when prescribed by an oncologist [24,25]. Exception status application can be requested by oncologists in Nova Scotia, New Brunswick, Newfoundland and Labrador (NL), Quebec, Yukon, and under NIHB. However, Alberta and BC do not include supportive care medications on their cancer formulary. In PEI, patients are encouraged to apply to the high-cost drug program if prescribed pegfilgrastim.

## 5. The Role of Patient Support Programs (PSPs)

The pharmaceutical industry plays an important role in cancer drug access within the Canadian system. Drug companies may open patient support programs (PSPs) to facilitate access to cancer medications. The financial coverage varies, but commonly includes co-pay support when provincial or private insurance does not cover the full cost of an oral medication [18]. Some PSPs will also provide free access to medications for patients, while a therapy is going through the HTA process or allow patients to access a cancer drug not available to them on public formulary, e.g., public coverage only indicated for patients over age 65. These programs are designed as temporary bridges to treatment and ultimately can be closed at the discretion of the providing company.

**Table 1:** Overview of public financial coverage of oral, intravenous (IV) cancer drugs and supportive care drugs by province, territory and federal insurance formularies. For supportive care drugs, we specifically looked at coverage for Neurokinin-1-receptor antagonists and pegfilgrastim. Jurisdictions may have different drugs on public formulary [24,26,27,28,29,30,31,32,33,34,35,36,37,38,39,40].

Jurisdiction	Oral Cancer Drugs	IV Cancer Drug	Supportive Care Drugs
Alberta	Covered, if on formulary	Covered, if on formulary	Not covered
British Colombia	Covered, if on formulary	Covered, if on formulary	Not covered
Manitoba	Covered, if on formulary	Covered, if on formulary	Exception Status
New Brunswick	Exception Status	Covered, if on formulary	Covered, if on formulary
Newfoundland and Labrador	Exception Status	Covered, if on formulary	Exception Status
Nova Scotia	Exception Status	Covered, if on formulary	Exception Status
Prince Edward Island	Limited coverage	Covered, if on formulary	Limited coverage
Ontario	Exception Status	Covered, if on formulary	Exception status
Saskatchewan	Covered, if on formulary	Covered, if on formulary	Covered, if on formulary
Quebec	Exception Status	Covered, if on formulary	Exception Status
Yukon	Exception Status	Covered, if on formulary	Exception Status
Non-insured Health Benefits, Northwest Territories Extended Health Benefits	Exception Status	Covered, if on formulary	Exception status

Green: For patients meeting drug indications, full public drug coverage is provided. Orange: Public drug coverage requires a patient to meet drug indications and an application for exception status. Blue: Public formulary provides full coverage for select medications. Red: Public coverage not offered.

## 6. Discussion

All Canadians expect and deserve equal access to cancer drugs. This narrative review documents disparities in access to cancer therapies across Canada, from differences between provincial and territory drug formularies, to gaps in coverage for THCDs. As a narrative review, this article is limited by the small sample of the literature included. The interpretation and discussion are influenced by the authors’ own perspective and biases.

At the provincial and territory level, the decision to add a drug to the public formulary is a closed process and the factors influencing these decisions are not well captured in the literature. Furthermore, Canada’s current health policies have led to a patchwork of variable coverage between jurisdictions for all cancer drugs, both IV and take home. Compared to other countries, Canada’s drug approval process takes longer and is associated with higher costs to patients and fewer drugs on market [41].

As a small market, Canada can consider adopting novel drug assessment methods, such as including real-world evidence [42], particularly for rare cancers or questions that are unlikely to be answered by randomized controlled trials. Preferentially opening post marketing phase IIIB/IV trials in cancer centres can serve dual purposes of providing a new route of drug access and allow for more data collection on health-related outcomes to support funding submissions. Alternative funding models may also provide solutions, including health outcomes-based models where manufacturers supply a new drug for a patient, and the public payer assumes cost only after clinical benefit is proven. Another option would be a mortgage model allowing payers to divide the cost of a drug over a period of time. Such an option would make a drug more affordable, especially for cancer diagnoses where there is only one indicated therapy that lacks direct market competition [43].

A number of economic analyses have reviewed the benefits of supporting patients’ access to THCDs, including Taylor in 2014 [17] and again more recently by CanCertainty [19]. THCDs are generally less costly to administer, not requiring chemotherapy chair time and necessary supporting staff to administer treatment [17]. Catastrophic drug programs in Ontario and Atlantic provinces offer support via processes that are excessively complicated and time consuming, often providing support too late for patients. They are only partially effective at covering drug costs; the resultant financial toxicity interferes with patients receiving the best possible cancer treatment [21].

A range of consequences have been reported by cancer patients experiencing financial toxicity, some of which can compromise their cancer care. Longo documented patients minimizing drug costs by rationing their medications or modifying treatment plans due to cost concerns (e.g., being admitted to hospital for inpatient treatment) [44]. Patients reported that finances play a role in their treatment decisions. From a societal perspective, patients should receive the therapy that best promotes their well-being, ability to contribute to society and generate less burden on the healthcare system and caregivers. In the era of precision oncology, that best therapy frequently is an oral THCD. Patients in Ontario and Atlantic Canada, especially under the age of 65, urgently need a simplified and fair process for accessing THCDs.

Considering solutions at a provincial level, the cancer agency model would offer Ontario and Atlantic provinces an approach to addressing inequity in THCD access. This model, in addition to reducing financial toxicity, concentrates patients with health care professionals with oncology expertise, for example, pharmacists with experiencing in Oncology. Cancer Care Ontario reported on the need to improve the safety and quality of THCD delivery [45]. A number of concerns were raised regarding dispensing of THCDs in community pharmacies, including incorrect dosing, limited monitoring, drug wastage, and increased dispensing cost to patients.

Quebec has taken a unique approach, mandating all adults in the province have drug coverage be it with the province or privately. No patient anticipates a cancer diagnosis and requiring coverage helps to mitigate sudden and unexpected financial burden. Quebec has successful raised insurance rates of working age individuals [46]. While this approach may be expected to reduce the cost to the government purse, 2019 THCD sales reveals 70% were covered still publicly [6].

Ultimately, the CHA requires similar standards of care be delivered to all Canadians, no matter their home address. Oncology has set a precedent in national healthcare, with harmonization of HTA through the pCODR process and CADTH. However, CADTH’s recommendations to provinces are not mandatory and, as such, differences in cancer drug formularies exist. CADTH is currently exploring the development of a pan-Canadian formulary. In the long-term, the development of a pan-Canadian oncology drug formulary, including selected cancer agents recommended by CADTH and requiring public coverage, could standardize access to THCDs [47].

## 7. Conclusions

Disparity in public coverage of cancer drugs, particularly THCDs, has led to inequities in the access to cancer drugs across Canada. While this is not a novel finding, oncology is increasingly using THCDs, and there is a growing urgency to address the financial toxicity faced by cancer patients in Canada. We urge policy makers in Ontario and the Atlantic provinces to consider adopting a cancer agency model. Nationally, the standards for access to cancer drugs could be set through the creation of a pan-Canadian oncology drug formulary.

Drug access is an important issue for patient advocacy groups to be aware of and to provide a united voice demanding policy reform. Finally, we would encourage ongoing health service research in this area to document the impact of disparities in drug access on the outcomes for Canadian cancer patients.

## Data Availability

Article library available on request.
